# Romantic relations, sexuality and intimacy among young adults and adolescents with severe mental illness: a review of the literature

**DOI:** 10.1186/s12888-025-07224-1

**Published:** 2025-11-26

**Authors:** Miriam Belluzzo, Camilla Esposito, Erica De Alfieri, Veronica Giaquinto, Daniela Volpe, Anna Lisa Amodeo

**Affiliations:** 1 Department of Mental, Physical Health and Preventive Medicine, University of Campania “Luigi Vanvitelli”, Largo Madonna delle Grazie, 1, Napoli, 80138 Italy; 2 SInAPSi Centre of University of Naples “Federico II”, Via Giulio Cesare Cortese, 29, Napoli, 80138 Italy; 3 Department of Humanities, University of Naples “Federico II”, Porta di Massa 1, Napoli, 80138 Italy

**Keywords:** Severe mental illness, Schizophrenia, Psychosis, Sexuality, Intimacy, Romantic relations, Young adults, Adolescents

## Abstract

**Background:**

Individuals with severe mental illness (SMI), including schizophrenia and psychotic disorders, often face significant challenges in developing and maintaining romantic and intimate relationships. Despite a strong desire for companionship and sexual expression, these individuals frequently encounter barriers such as social stigma, cognitive issues, and psychiatric symptoms affecting social functioning. Existing research primarily focuses on general social engagement rather than the specific complexities of romantic and sexual relationships. This narrative review synthesizes recent literature (2017–2023) to explore the intersection of SMI with sexuality, intimacy, and romantic relationships among adolescents and young adults.

**Methods:**

A narrative review approach was used to examine studies published in English between January 2017 and December 2023. Literature searches were conducted using Web of Science and Scopus, with Boolean operators applied to keywords related to severe mental illness, adolescence and young adulthood, sexuality, and romantic relationships. Inclusion criteria focused on studies addressing intimacy, romantic relationships, and sexuality among individuals diagnosed with schizophrenia or psychosis. Studies that primarily discussed medical, forensic, or legal aspects without addressing relational or psychological dimensions were excluded. The selected literature was analyzed thematically.

**Results:**

Five key themes emerged: (1) Sexual risk-taking behaviors, highlighting increased vulnerability to unprotected sex, sexually transmitted infections (STIs), and exploitation; (2) Sexual dysfunctions, often related to psychiatric symptoms and medication side effects; (3) Personal and relational resources, including the role of self-esteem, social cognition, and relationship quality in supporting romantic engagement; (4) Social stigma, which impacts self-perception, desirability, and access to relationships; and (5) Sexual orientation and gender identity, emphasizing the unique challenges faced by LGBTQ + individuals with SMI, who experience compounded stigma and relational difficulties.

**Conclusions:**

This review highlights the unmet needs for intimacy, love, and sexual expression among individuals diagnosed with SMI and the significant barriers they face. Findings suggest that psychosocial interventions, comprehensive sexual education, and stigma reduction strategies are essential for improving relationship opportunities and overall well-being. Future research should focus on lived experiences and explore tailored interventions to support romantic and sexual relationships in this population.

**Supplementary Information:**

The online version contains supplementary material available at 10.1186/s12888-025-07224-1.

## Background

Severe mental illness (SMI) refers to a group of psychiatric diagnosis defined as “mental, behavioral, or emotional disorders resulting in serious functional impairment, which substantially interfere with or limits one or more major life activities” [1]. For the purposes of this manuscript, SMI includes conditions such as schizophrenia, schizoaffective disorder, and psychotic disorders, all of which are characterized by long-term or recurring symptoms, including delusions, hallucinations, cognitive issues, and impaired emotional regulation. These diagnoses often lead to serious functional challenges that impact not only cognitive processes but also an individual’s social, occupational, and personal life. As a result, individuals diagnosed with severe mental illness (DSMI) face substantial challenges in forming and maintaining intimate and romantic relationships, as well as experiencing sexuality in a meaningful and fulfilling way [2, 3].

Despite advancements in treatment, individuals with psychosis still face substantial challenges in building meaningful social connections [4–7].

Individuals diagnosed with severe mental illnesses, especially during early and young adulthood, express a strong desire for love and companionship, despite the barriers posed by their condition. However, many experience social isolation and difficulty sustaining romantic relationships, leading to smaller social networks and greater reliance on service providers [4, 8]. Gender differences also play a role, with young men often facing more significant challenges in dating and social functioning compared to women, possibly due to the more severe onset of psychosis in males [9]. Additionally, marital status may affect illness progression differently by sex, with symptoms exacerbated in women and alleviated in men, possibly due to differing patterns of marriage retention based on symptom severity [9, 10].

Several factors contribute to difficulties in romantic engagement, including challenges in social functioning, neurocognitive differences, psychiatric symptoms, stigma, and anticipated discrimination [8, 11–13]. In particular, low or fluctuating self-esteem, insecure attachment styles, and challenges in social functioning further hinder individuals DSMI in initiating and maintaining romantic relationships [14]. The fear of rejection and insecurity create additional barriers to forming lasting connections, while missed developmental milestones, such as disrupted education and employment, exacerbate these issues [12–15].

Despite the recognized importance of romantic relationships for individuals diagnosed with SMI, research has largely focused on general social engagement rather than specific factors influencing romantic relationships. This gap limits the development of tailored psychosocial interventions that address the unique challenges faced by this population [16, 17].

This study presents an overview of the most recent literature (2017–2023) on sexuality, intimacy, and romantic relationships in adolescents and young adults diagnosed with SMI, with a focus on schizophrenia and psychosis. The aim is to inform future interventions that meet the real needs of this population. Addressing the challenges in forming and maintaining romantic relationships is essential for enhancing the well-being and social integration of young individuals diagnosed with SMI.

## Methods

The review includes literature on intimacy and sexuality among young adults or adolescents with schizophrenia, psychotic disorders, or, more generally, with severe mental illness as participants. We selected only articles in English, published between January 2017 and December 2023, to capture the most recent and relevant information on the topic. This time frame was chosen to ensure the inclusion of studies reflecting the latest developments and trends in the field while maintaining relevance to the focus of the current research.

It is important to note that the term “young adults” does not have a universally agreed-upon definition; its meaning varies according to the research context. In psychological and sociological literature, for instance, the concept of “emerging adulthood”—typically referring to the age range of 18 to 25 years as described by Arnett [18]—is frequently employed.

From a psychological perspective, however, the concept of “young adulthood” is further shaped by developmental theories, such as Erikson’s stages of psychosocial development [19, 20], which situate the key acquisitions associated with this life stage within a broad age range spanning late adolescence to approximately 40 years of age [21, 22]. These models suggest that the transition into adulthood is influenced not only by chronological age but also by critical developmental achievements in areas such as maturation, occupational status, sexual identity, and emotional regulation.

Considering the unique developmental trajectories and clinical profiles of people diagnosed with severe mental illness [23], our review adopts a broader definition, including studies focusing on participants up to about 40 years of age or studies whose average ages of the participants is around 40 years. This decision is based on the evidence that the onset and progression of diagnoses such as schizophrenia often extend beyond the frequently employed definition of early adulthood period [18] and that, therefore, early interventions to support this population must take these specificities into account [23]. Expanding the age range allows for a more comprehensive exploration of the evolution of intimate and romantic relationships in the context of the ongoing psychosocial and clinical challenges associated with severe mental illness.

The electronic databases used were Web of Science and Scopus. Searches were performed using Boolean operators (AND/OR) with the following search string applied to titles, abstracts, and keywords:

*(“Down syndrome” OR schizophrenia OR psychosis OR “mental disability” OR “mental illness”) AND (adolescence OR “young adult” OR “young adults” OR “teenager”) AND (sexuality OR intimacy OR love OR romance OR romantic).*

**Fig. 1:**
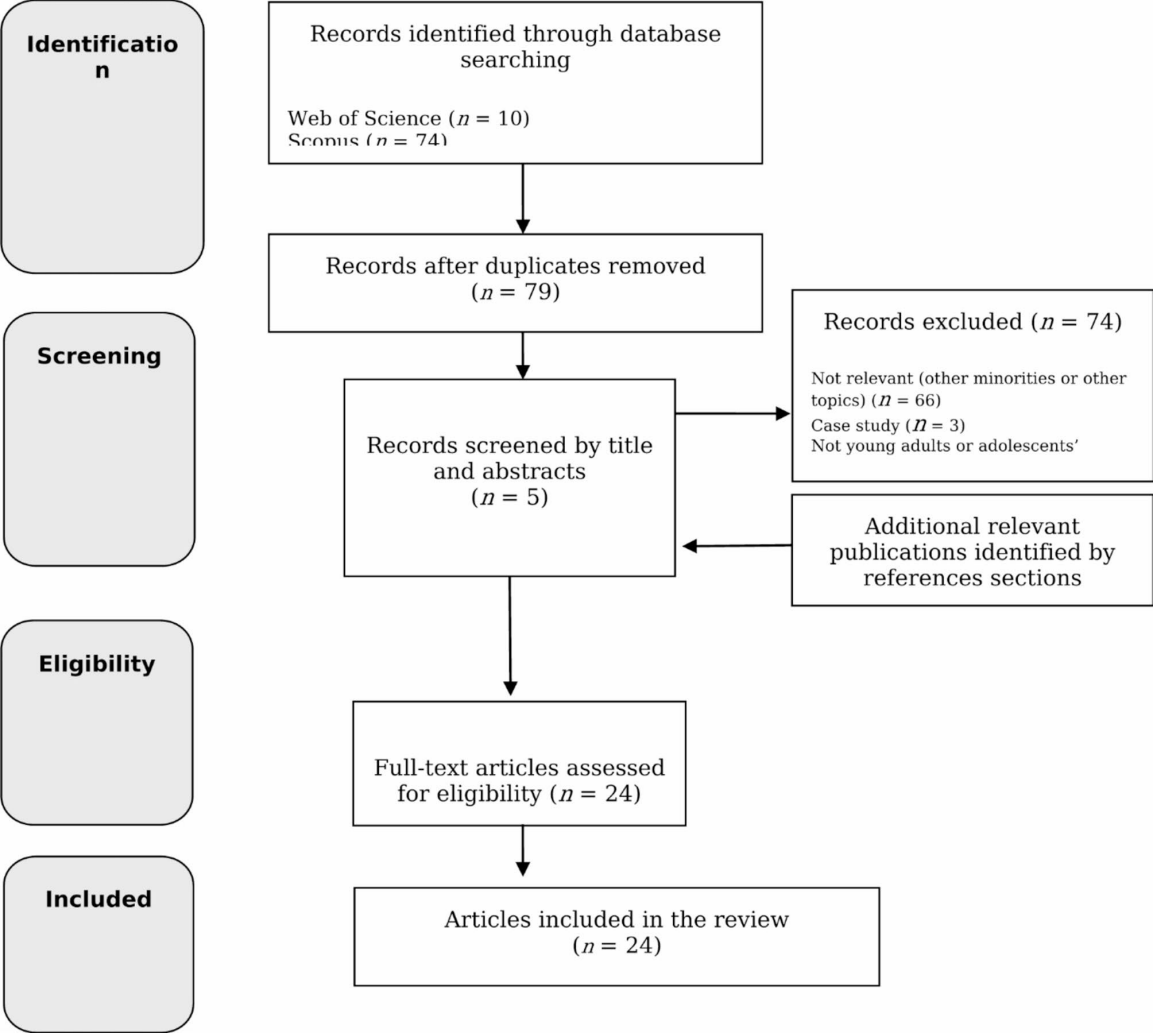
Flowchart of the search results

After an initial search of each database, a total of 84 potential records were identified and exported to Zotero, with 5 sources eliminated as duplicates. The remaining 79 sources were screened by title and abstract. Case studies, books, letters, opinion papers, commentaries, and articles that did not have individuals with severe mental illness as participants or that primarily focused on medical or juridical issues with only a marginal psychological impact were excluded. The reference sections of the included articles were reviewed for additional relevant publications that may have been missed. The search results are presented in a flowchart (Fig. 1).

Although “Down syndrome” was included in the original search string for the sake of completeness, no studies focusing specifically on this population were retained in the final synthesis studies. This decision was made to ensure conceptual consistency, as the specific cognitive and developmental profiles of individuals with Down syndrome differ significantly from those typically associated with serious mental illness (SMI), which was the primary focus of the review.

The selection process involved MB, CE, EDA, VG, and DV, who independently screened the titles and abstracts of the identified studies. In cases of disagreement, ALA was consulted to resolve discrepancies. All authors then reached a consensus on the inclusion of the final studies by discussing the relevance and quality of each article. This collaborative process ensured consistency in the selection criteria and helped minimize bias in study selection. At the end of the process, 24 studies were included in the review. The relevance of the studies was determined based on their focus on intimacy, sexuality, and romantic relationships among adolescents and young adults diagnosed with severe mental illness (SMI), specifically schizophrenia and/or psychotic diagnoses, provided they explicitly targeted this population.

A qualitative thematic analysis was conducted to synthesize the key themes and insights across the selected studies, offering a nuanced understanding of the challenges faced by individuals diagnosed with SMI in the realms of sexuality and romantic relationships. This method was chosen because it allows for a structured yet flexible synthesis of qualitative findings across diverse studies, facilitating the identification of recurring patterns, conceptual relationships, and key challenges related to intimacy and sexuality.

The synthesis followed the framework outlined by Braun and Clarke [24], employing inductive thematic analysis to identify common themes across the studies. The analytical process involved the following steps:

- *Familiarization with the data*– All included studies were read multiple times to identify recurring patterns and key insights.
- *Initial coding*– Relevant findings were systematically extracted and assigned descriptive codes related to relational and sexual experiences.
- *Identification of thematic patterns*– The extracted codes were grouped into broader themes that captured shared concepts across studies.
- *Review and refinement*– Themes were refined to ensure coherence and distinctiveness, eliminating overlaps or ambiguities.
- *Final synthesis*– Themes were named, defined, and interpreted in relation to existing literature and theoretical frameworks.

To increase transparency and rigor, two researchers (MB and CE) independently coded the data. Five consensus meetings were held to discuss emerging themes, refine the coding scheme, and resolve any discrepancies. Another author (ALA) participated in these meetings to provide additional insights and ensure that all interpretations were grounded in the data. This iterative process helped reduce bias and strengthened the reliability of the thematic synthesis.

Moreover, to provide a comprehensive synthesis of the findings, a summary table (Table 1) was included at the end of the Results section.

From the analysis of the results of the selected studies, several key themes emerged: (1) Sexual risk-taking behaviors– Including unprotected sex, vulnerability to sexual exploitation, and sexually transmitted infections (STIs); (2) Sexual dysfunctions– Associated with psychiatric symptoms, medication side effects, and psychosocial factors; (3) Personal and relational resources– Encompassing self-esteem, social skills, and the role of supportive relationships in fostering intimacy; (4) Social stigma– Internalized and external stigma, shaping self-perception, desirability, and access to romantic relationships; (5) Sexual orientation and gender identity– Addressing the challenges faced by LGBTQ + individuals with SMI, including compounded stigma and relational difficulties.

Each of these themes will be explored in further detail in the Results section.

## Results

Schizophrenia and related psychotic diagnoses are defined in the DSM-5 [25] as conditions involving abnormalities in one or more of the following five domains: delusions, hallucinations, disorganized thinking (speech), grossly disorganized or abnormal motor behavior (including catatonia), and negative symptoms. These conditions represent a heterogeneous group, with varying symptom severity, which can predict significant aspects of the illness such as cognitive or neurobiological deficits, the potential for remission, and the impact on the overall quality of life [25]. The prevalence of psychotic syndromes is estimated at around 0.2%, with the persecutory subtype of delusional disorder being the most common, and they can occur in both young people and adults [25]. According to Larson et al. [7] and the DSM-5 [25], schizophrenia is characterized by disassociation from reality (psychosis), auditory hallucinations, delusions, atypical thinking and behavior, limited emotional expression, decreased motivation, cognitive deterioration, and impairment in daily activities such as work, social interactions, and self-sufficiency [7, 25, 12; 13]. This condition typically manifests at a young age and can lead to significant disability and long-term social challenges. Schizophrenia is considered one of the most severe clinical conditions, given its psychological, social, and economic impacts on human life.

Individuals diagnosed with severe mental illnesses often face human rights violations, and the stigma associated with these disorders is widespread in society. This stigma is frequently internalized, which has a profound effect on their self-esteem and their ability to form meaningful emotional relationships, crucial for the expression of their sexuality [12, 15, 26, 27]. Consequently, they may experience marginalization, further exacerbating the challenges they encounter in their social interactions, which are already strained by the symptoms of their illness [15]. Those diagnosed with serious mental illness are more likely to be isolated, with poorer social networks compared to the general population. This isolation stems from both the psychotic symptoms, which make social interactions difficult, and the pervasive social stigma [9, 12, 13].

Numerous studies [12, 17, 28] have highlighted that, like the general population, individuals DSMI have unrecognized and unmet needs for intimacy, love, and sexuality, even though their desires, fantasies, and need for romantic relationships are not significantly different from those of the general population. Moreover, having a fulfilling emotional and romantic life is positively correlated with better prognosis and quality of life [16, 17]. However, the sexual and reproductive health of individuals diagnose with SMI, particularly during adolescence and young adulthood, is notably poorer than that of the general population, with multiple interrelated factors contributing to this disparity [12, 29]. Evidence suggests [2, 15, 27, 30, 31] that, for young people living with a severe psychiatric diagnosis, the unmet right to sexual and reproductive health increases vulnerability to gender-based violence, sexual risk-taking behaviors (such as unprotected sex), and a higher risk of sexually transmitted infections (STIs), unwanted pregnancies, and abortions. Additionally, these individuals face increased vulnerability to sexual coercion, assault, and sexual exploitation, particularly when coming from socially and economically marginalized backgrounds.

Furthermore, several specific dimensions of psychotic symptomatology must be considered. Illness, particularly in people living with severe mental illness (PLSMI), often represents a turning point, challenging the identity these individuals held before, and straining their interpersonal relationships [5, 28]. A cyclical process exists where self-stigmatization fosters self-isolation, leading to decreased social functioning and amplifying stigma, which negatively affects the emotional and sexual aspects of these individuals’ lives. Additionally, deficits in emotional, social, and cognitive skills are common in the psychotic and schizophrenic spectrum diagnosis, often manifesting as a loss of one’s sense of self, trust in one’s own memories and feelings, and trust in others [12, 13, 32].

### Sexual risk-taking behaviors

Research consistently indicates that young people with a severe psychiatric diagnosis, particularly those with psychosis and schizophrenia, engage in risky sexual behaviors at higher rates than their non-psychotic peers and the general population [29–31, 33]. These behaviors include unprotected sex, multiple partners, unplanned pregnancies, and increased vulnerability to sexually transmitted infections (STIs), including HIV. The underlying factors contributing to these behaviors are complex and multidimensional, involving both individual and environmental influences.

A key factor influencing sexual risk-taking is the interaction between psychotic symptomatology and social functioning. Some studies suggest [27, 29–31, 33] that individuals with moderate psychotic symptoms and sufficient social skills are more likely to engage in sexual relationships, but their ability to assess risks and adopt protective strategies may still be compromised. For instance, Brown et al. [27] found that among young people experiencing first-episode psychosis, a significant proportion reported being sexually active, yet two-thirds of these individuals did not use condoms regularly, and more than one-fifth had experienced an unplanned pregnancy. Notably, sexual activity was more frequent among individuals who consumed substances, highlighting the role of substance use as a key factor in increasing exposure to high-risk sexual encounters. Vickers et al. [30] confirmed these patterns, emphasizing that young people diagnosed with psychosis were more likely to engage in unprotected sex, have multiple partners, and use drugs before intercourse. The increased likelihood of contracting STIs in this population suggests a significant gap in sexual health education and preventive care.

Beyond general risk-taking behavior, some studies highlight [27, 30, 33] the impact of psychotic symptomatology on individuals’ awareness of their own sexual boundaries. During the positive phase of psychosis, a substantial proportion of individuals report an increase in sexual desire, which, in some cases, is accompanied by a reduced ability to recognize personal limits and communicate needs effectively. De Jager et al. [33] found that 38% of participants experiencing psychosis reported increased sexual desire, with 18% simultaneously experiencing diminished awareness of their boundaries. This dynamic places individuals at heightened risk of engaging in sexual activity in unsafe or non-consensual contexts, further exacerbating their vulnerability to exploitation and coercion.

Gender differences also emerge as a critical factor in sexual risk-taking within this population. Schonewille et al. [29] found that among young women diagnosed with severe mental illness, unplanned pregnancies were a common occurrence. The risk factors contributing to this issue extend beyond impaired decision-making and lack of contraception use to include experiences of victimization, sexual coercion, and exposure to violence. This suggests that the vulnerabilities faced by women DSMI are not solely a function of individual behavior but are also shaped by broader social and structural inequalities. Additionally, Adan-Sanchez et al. [31] observed that 26% of young people diagnosed with psychosis attending a mental health service had experienced a pregnancy, and 95% of these pregnancies were unplanned. Their study highlighted that non-consensual sex and substance use were strongly associated with high-risk sexual behaviors and adverse reproductive outcomes, reinforcing the findings of previous research.

Another important consideration is the role of symptom severity in influencing sexual behavior. While it might be assumed that more severe psychiatric symptoms correlate with greater sexual risk-taking, some studies indicate otherwise. Adan-Sanchez et al. [31] found that the severity of mental health symptoms was not directly linked to an increase in risky sexual behavior. Instead, social factors—such as substance use, history of sexual violence, and overall life stability—appeared to be stronger predictors. This finding challenges simplistic assumptions about the relationship between mental illness and sexual health and suggests the need for a more nuanced understanding of the diverse experiences of individuals DSMI. Comprehensive sexual health education tailored to this population, as well as support programs aimed at promoting safe and consensual sexual relationships, should be considered essential components of mental health care. Furthermore, addressing the structural vulnerabilities faced by young women diagnosed with SMI, including their increased exposure to victimization, should be a priority in both clinical and policy-level interventions.

### Sexual dysfunctions

In accordance with the DSM-5 [25] “sexual dysfunctions” can be defined as clinically significant disturbances in an individual’s ability to experience sexual desire, arousal, or orgasm, or the presence of pain during sexual activity, which cause marked distress or impair interpersonal relationships. It is important to note that a mere lack of romantic or sexual interest is not necessarily considered a sexual dysfunction unless it results in significant personal distress or functional impairment.

Sexual dysfunction is a prevalent yet often overlooked issue among individuals diagnosed with severe mental illness (SMI). While much of the literature focuses on the biological and medical dimensions of this condition, a growing body of research [28, 34, 35] highlights the interplay between psychotropic medication side effects, symptoms of mental illness, and socio-environmental factors in shaping individuals’ sexual experiences and difficulties. The consequences of these dysfunctions extend beyond physical symptoms, affecting self-esteem, relationship dynamics, and the capacity for intimacy.

The systematic review by McCann et al. [34] underscores the multifaceted nature of sexual dysfunctions in individuals DSMI, noting that decreased libido, difficulty achieving orgasm, and feelings of inadequacy are frequently reported. These impairments contribute to dissatisfaction in sexual relationships and, in some cases, lead to avoidance of intimate connections.

Another significant contributor to sexual dysfunction in this population is the impact of psychiatric medications. Kaplan et al. [35] emphasize that many patients experience reduced sexual desire, erectile dysfunction, and premature ejaculation, which in turn negatively affect self-perception and masculinity. Despite these well-documented side effects, medical professionals often fail to address them adequately in clinical practice. The lack of proactive discussions about potential sexual side effects during the prescription process underscores a critical gap in psychiatric care.

While pharmacological factors play a crucial role, research indicates that sexual dysfunction in individuals with psychotic symptoms is not solely attributable to medication use. Vickers et al. [30] and Barchielli et al. [28] highlight that many young adults and adults with psychotic diagnoses experience persistent sexual dysfunction independent of their pharmacological treatment. Barchielli et al. [28], in particular, stress the importance of adopting a biopsychosocial approach to understanding sexual difficulties in this population. Their study, which compared 46 individuals diagnosed with psychosis to a control group of 52 healthy individuals, found that those with psychosis reported significantly higher rates of sexual dysfunction, including premature ejaculation, diminished orgasm satisfaction, and decreased sexual desire and arousal, particularly among individuals diagnosed with schizophrenia.

De Jager et al. [34] further illustrate the complexity of sexual dysfunction in individuals diagnosed with SMI by exploring how psychotic episodes influence sexual experiences. In their study, 14% of participants reported a decrease or complete absence of sexual drive during psychotic episodes, while others described disruptions in ejaculation and orgasm that appeared to be linked to changes in intimacy and relational distance. Interestingly, some participants reported that overcoming these difficulties, combined with therapeutic insights, led to increased openness and trust toward others, thereby improving their overall capacity for sexual expression.

Beyond pharmacological and symptomatic explanations, Fanta et al. [36] argue that psychological factors related to medication side effects, such as obesity and prolactin secretion, significantly influence individuals’ willingness to engage in social and sexual relationships. In a study involving 422 patients diagnosed with schizophrenia, those who were single, divorced, or widowed exhibited higher rates of sexual dysfunction compared to those in committed relationships. Furthermore, individuals with a history of recurrent relapses were three times more likely to experience sexual dysfunction, suggesting that disease progression and the predominance of negative symptoms—such as avolition, anhedonia, and blunted affect—play a crucial role in shaping sexual health outcomes.

A common theme across multiple studies is the difficulty individuals with psychotic diagnoses experience in managing intimacy and close relationships. Barchielli et al. [28] emphasize that these difficulties often result in avoidance behaviors and self-victimization, which further limit opportunities for emotional and sexual connections. Rather than being solely a side effect of medication or a symptom of the illness, sexual dysfunction in individuals diagnosed with SMI appears to be deeply intertwined with their broader relational and social experiences. These findings reinforce the argument that psychosis itself fosters vulnerability in sexual expression, positioning sexual dysfunction as both a consequence and a reinforcing factor in the broader spectrum of relational impairments associated with severe mental illness [3, 28, 30].

Taken together, these studies highlight the necessity of moving beyond a purely medicalized perspective when examining sexual dysfunction in individuals diagnosed with SMI. A comprehensive understanding requires acknowledging the interaction between biological, psychological, and social dimensions, ensuring that both clinical and environmental factors are considered in interventions aimed at improving sexual well-being in this population.

### Personal and relational resources

An emerging body of research [3, 9, 11, 13, 16, 35, 37] underscores that personal and relational resources are critical to the establishment and maintenance of healthy romantic and sexual relationships among individuals diagnosed with severe mental illness (DSMI). Rather than viewing these findings as isolated reports, the literature collectively suggests that factors such as self-esteem, illness acceptance, social cognition, and supportive interpersonal relationships interact to influence sexual well-being.

For instance, Gerymski and Szelag [37] explored the interplay between sexual well-being, illness acceptance, and self-esteem among 60 Polish individuals diagnosed with schizophrenia (SCH). Their pilot study revealed that accepting one’s illness was closely linked to a positive self-attitude and better sexual well-being. Individuals who embraced their limitations were more likely to report higher levels of physical, emotional, and social well-being, which in turn promoted healthier sexual desire and satisfaction. Notably, the study also highlighted gender differences—men demonstrated higher self-esteem than women, possibly due to a more unfavorable illness trajectory in women—which suggests that personal resources can mitigate some of the negative impacts of schizophrenia on intimate relationships.

Sexual expression itself plays a fundamental role in constructing one’s identity and self-esteem. De Jager et al. [33] found that although a high percentage of individuals diagnosed with psychosis (83%) experience sexual feelings, many struggle to effectively express their sexuality. Impaired social cognition and communication were identified as key barriers, with 32% of participants reporting difficulties discussing sexual topics with partners and 46% of unmarried individuals facing challenges in accessing potential partners. These difficulties underscore that deficits in interpersonal skills can hinder the expression of sexuality, thereby impacting overall well-being.

Further supporting these observations, Stusiński et al. [9] reviewed the challenges faced by people diagnosed with schizophrenia in initiating and maintaining dyadic relationships. Their analysis revealed that despite having unaltered relational needs, individuals diagnosed with SCH often experience social withdrawal, a diminished “ability to love,” and poorer relationship quality. Interestingly, gender differences emerged again, with women generally faring better in forming intimate relationships—possibly due to a later onset of the illness that allows the development of social skills prior to its active progression. The review also noted that successful intimate relationships could serve as a source of emotional support and motivation, aiding recovery and improving overall functioning.

Complementing these findings, White et al. [16] demonstrated that satisfaction within romantic relationships is significantly associated with improved well-being outcomes among people diagnosed with psychosis. Their study of 232 individuals showed that partner criticism was linked to lower relationship satisfaction, while positive, supportive interactions were connected with reduced psychotic symptoms and higher self-esteem. In this way, a satisfying romantic relationship can buffer against loneliness and internalized stigma, reinforcing a positive identity that extends beyond the illness.

Moreover, McCann et al. [34] emphasize the role of supportive relationships—both familial and romantic—in the recovery process. They argue that involving partners in treatment and providing robust psychosocial support can help counteract the stigma, self-stigma, and isolation frequently experienced by individuals diagnosed with SMI. Similarly, the meta-synthesis by Hortal-Mas et al. [11] illustrates that the sexuality of people living with serious mental illness (PLSMI) is mediated by challenges in understanding nonverbal cues, intrusive symptoms, and social isolation. Such challenges may lead to feelings of loneliness, insecurity, and shame, which in turn can hinder intimate relationships and sexual expression.

Qualitative studies further reinforce these themes. De Jager et al. [3] observed that the absence of a partner was a primary source of dissatisfaction with intimacy for many individuals diagnosed with psychosis. Conversely, when relationships were characterized by mutual understanding and stability, they not only provided emotional support but also facilitated a clearer separation of mental illness from one’s overall identity. However, when both partners struggled with mental health issues, relationship dynamics sometimes became imbalanced, limiting each partner’s ability to fully engage in intimacy.

Additional evidence comes from Cloutier et al. [13], who identified low social functioning—including challenges in independent living and maintaining relationships—as significant barriers to forming romantic ties among individuals diagnosed with schizophrenia-spectrum disorders. Finally, practical issues such as the lack of private spaces, limited dialogue about sexuality with staff, and insufficient protective measures (e.g., condoms) further complicate the ability of patients to express their sexuality in a healthy manner [35].

The literature indicates that personal and relational resources—including self-esteem, illness acceptance, social communication skills, and the quality of supportive relationships—are integral to the sexual well-being of individuals diagnosed with SMI. These resources not only mitigate the direct impacts of psychiatric symptoms but also help counterbalance environmental and social challenges. By integrating these dimensions, interventions can be designed to promote healthier sexual expression and more satisfying intimate relationships, ultimately contributing to improved recovery and overall quality of life.

### Social stigma

Social stigma plays a key role in the difficulties faced by individuals diagnosed with psychosis spectrum in the realm of sexuality and relationships. The social labeling of mental illness negatively influences the perception of these individuals as desirable sexual partners, contributing to reduced self-confidence and self-efficacy in fundamental areas of human experience such as intimacy and sexuality [26]. Stigmatization is not only an external phenomenon but is often internalized by individuals diagnosed with SMI, leading to the construction of an identity centered on illness. White et al. [16] found that individuals diagnosed with psychosis tended to perceive themselves as undesirable due to their condition, with self-stigma being correlated with greater severity of psychiatric symptoms.

A large-scale study by de Jager et al. [33] revealed that 68% of participants had experienced public stigma related to their psychosis, with more than one-third facing rejection from friends and sexual partners following a psychotic episode or after disclosing their diagnosis. The internalization of this stigma led many to perceive themselves as vulnerable and undesirable, resulting in significant sexual and relational exclusion: 29% of participants had completely avoided intimate relationships for over seven years. Moreover, the same study highlighted how concerns about symptoms or medication side effects—such as sweating and tremors—increased social anxiety and the fear of being perceived negatively by others. This generated profound insecurity in social interactions, with some participants reporting a fear of “doing something wrong”, leading them to avoid contact with potential partners altogether. The fear of judgment and past experiences of discrimination often resulted in a vicious cycle of isolation and distrust towards others, with a reduced willingness to disclose their condition unless a strong bond of trust was present [3].

Another dimension of stigma affects women diagnosed with SMI, as analyzed by Grachev et al. [26] in their literature review on female sexuality in the context of severe mental illness. Their analysis highlighted how stigma influences women diagnosed with SMI at both a microsocial level, in daily interactions, and at a structural level, through institutions that limit their sexual and reproductive rights. In relationships, many women face the difficult choice of whether to disclose their diagnosis to partners, fearing rejection or relationship breakdown. Their perception of desirability is deeply affected, leading to lower self-esteem and a sense of being unfit for marriage or stable relationships. Additionally, these women experience a dual stigma, often being considered unfit for motherhood and perceived as potentially dangerous to their children, forcing them to constantly prove their parental competence. Beyond the psychological effects of stigmatization, the review emphasized that women diagnosed with SMI are at higher risk of sexual abuse and exploitation—both by strangers and partners—compared to the general population. Their economic vulnerability and dependence on psychotropic medications can further contribute to situations of coercion, forcing them to endure violent relationships or engage in unwanted, sometimes unprotected, sexual encounters [26].

Social stigma and self-stigmatization also have a significant impact on the experiences of individuals diagnosed with schizophrenia. Budziszewska et al. [32] found that 27% of participants had experienced discrimination, while 55% anticipated facing it in the future. The belief that “no one wants to have a schizophrenic partner” was internalized by many, leading to deep skepticism about the possibility of engaging in romantic relationships. The absence of romantic relationships was perceived as an obstacle to the recovery process, yet at the same time, psychosis itself was seen as incompatible with love. Furthermore, the economic difficulties often associated with schizophrenia represented an additional deterrent to forming relationships, highlighting an intersection between social stigma and economic stigma. Particularly critical was the risk of abuse for women with this diagnosis, a risk often underestimated and inadequately addressed in clinical and institutional settings.

An additional form of stigmatization emerges within psychiatric institutions themselves, where patients’ sexuality is frequently ignored or deemed inappropriate. Kaplan et al. [35] found that individuals hospitalized in psychiatric wards often felt that their sexual needs were overlooked or repressed, with healthcare staff systematically avoiding discussions about sexuality. This institutional silence not only amplified feelings of shame and inadequacy among patients but also led them to hide their sexual desires or abstain from discussing them, further reinforcing the taboo surrounding sexuality in individuals with mental diagnoses. In culturally conservative contexts, such as among the Arab Bedouin participants in the study, this stigma overlapped with pre-existing cultural norms, creating an additional layer of exclusion and repression.

Finally, it is important to consider how stigma disproportionately affects those who do not conform to traditional heterosexual relationships. Individuals diagnosed with SMI who belong to sexual and gender minorities experience a dual stigma, facing marginalization both for their gender identity or sexual orientation and for their psychiatric condition. This double stigma can increase feelings of isolation and create additional barriers to accessing safe spaces where they can explore their sexuality and identity [34].

Social and internalized stigma significantly hinder the expression of sexuality and the formation of intimate relationships for individuals diagnosed with SMI. The interplay of societal prejudice, institutional discrimination, and personal fears often leads to avoidance of romantic or sexual involvement, exacerbating feelings of loneliness and reducing overall quality of life. Women and non-heterosexual individuals face compounded stigma, as their psychiatric conditions intersect with gender and sexual orientation-related discrimination. Addressing these barriers requires increased awareness in clinical and social settings, fostering inclusion and self-determination in the affective and sexual lives of PLSMI.

### Sexual orientation and gender identity

The intersection of sexual orientation, gender identity, and mental health has been a complex and evolving area of focus, particularly in the context of individuals diagnosed with severe mental illness and psychiatric research. However, recent studies [11, 15, 34, 38] increasingly highlight the complex relationships between these aspects, particularly among LGBT + individuals and those with gender-nonconforming identities who experience serious psychiatric conditions. While psychosis and other severe mental health issues can influence self-perception, gender identity, and sexual orientation, the precise mechanisms of these interactions are still being elucidated [15, 17, 33, 38–40]. This section synthesizes findings from multiple studies to provide a more comprehensive understanding of how gender and sexual identity intersect with mental illness, particularly psychosis, and how these intersections are shaped by stigma, self-perception, and social factors.

A key theme emerging from the literature is the destabilizing effect of psychosis on gender and sexual identity. Several studies emphasize that during psychotic episodes, when individuals experience intense positive symptoms like hallucinations and delusions, the boundaries of gender and sexual orientation can become fluid or blurred [15, 17, 33, 33, 38]. For instance, de Jager and McCann’s [17] review points out that psychosis can lead to a loosening of these boundaries, with individuals sometimes engaging in behaviors or experiencing feelings—such as same-sex attraction—that may not align with their identity when well. This effect is particularly notable during the recovery phase, when the stabilization of psychotic symptoms often gives rise to confusion or shame regarding behaviors or attractions experienced during the episode. The findings from de Jager et al. [33] further reinforce this, showing that 18% of participants reported doubts about their sexual orientation, and 7% about their gender identity, after experiencing psychosis. The unstable self-perception during psychotic episodes often contributes to a lingering sense of confusion, sometimes leading individuals to question or reject parts of their identity after the psychosis subsides.

Further analysis of gender and sexual identity within the context of psychosis is provided by Barker et al. [15]. In their study of 19 participants—including cisgender women, transgender women, and non-binary individuals—they observe that psychosis can intersect with gender identity and sexual orientation in different ways. While some participants experienced these aspects of their identity as separate from their psychosis, others found them to be intertwined, with psychotic experiences exacerbating self-doubt. This theme of self-doubt reflects a broader pattern observed in the literature, where psychosis disrupts not only reality testing but also the sense of self, particularly in relation to intimate and sexual identity. The study suggests that psychosis, by undermining self-reliability, can lead individuals to question the authenticity of their gender and sexual identities, an issue that resonates with broader questions of self-perception in the context of SMI.

Additionally, the stigma surrounding both psychiatric illness and LGBT + identity compounds the challenges faced by individuals navigating both mental health matters and non-normative gender or sexual identities. Hortal-Mas et al.’s [11] meta-synthesis highlights the compounded stigma experienced by LGBT + individuals with severe psychiatric diagnoses. They found that the intersection of these two stigmatized identities often leads to greater social isolation, as individuals may feel the need to hide either their mental illness or their sexual orientation to avoid rejection. This double stigma can lead to further marginalization in healthcare settings, where individuals often report feeling dehumanized or misrepresented due to a lack of understanding among providers. This aligns with the findings of McCann et al. [34], who emphasize the need for mental health professionals to be more attuned to the specific challenges faced by LGBT + individuals diagnosed with SMI. These studies underscore the importance of addressing both the severe mental illness diagnoses and sexual identity of individuals in a holistic manner, which is often overlooked in traditional psychiatric care.

While an exploration of the historical and theoretical shifts in the pathologization of sexual orientation and gender identity provides important context for understanding the intersectionality of these identities with SMI, a more in-depth analysis of this issue would require a separate work, as it goes beyond the scope of this review. Here, the focus is primarily on recent studies and how they highlight the complexities surrounding sexual orientation and gender identity in individuals diagnosed with SMI. The historical context is briefly mentioned because understanding these shifts is essential for framing contemporary issues, but a full exploration of the pathologization of these identities would necessitate a dedicated work on its own. For example, Drescher [41] provides a historical perspective on the pathologization of homosexuality and gender variance within psychiatric classifications, noting the significant shift in 1973 when homosexuality was removed from the Diagnostic and Statistical Manual of Mental Disorders (DSM). Suess Schwend’s [42] work on the depathologization of transgender identities further emphasizes the importance of removing gender incongruence from the realm of mental illness, advocating for healthcare systems that respect and affirm diverse gender expressions and identities. Robles et al. [43] reinforce this shift by documenting the removal of gender variance from the DSM and ICD, with the inclusion of “gender incongruence” in a separate category outside of mental disorders, reflecting a growing recognition of the social and cultural factors that influence gender identity. These historical shifts are fundamental for understanding how gender and sexual identities have been framed within psychiatric contexts, but an extensive discussion of this evolution requires further examination beyond the scope of this review.

The reviewed studies collectively highlight the nuanced and complex relationship between psychosis, gender identity, and sexual orientation. Psychosis can destabilize gender and sexual identity, leading to confusion, shame, or self-doubt. However, it can also provide opportunities for self-exploration and transformation. The compounded stigma faced by LGBT + individuals diagnosed with SMI further complicates their experience, often leading to greater social isolation and challenges in healthcare settings. These findings suggest that mental health care providers must adopt a more inclusive and person-centered approach, one that recognizes the intersectionality of severe mental illness, gender, and sexual identity. Further research is needed to understand the broader implications of these intersections, particularly in the development of treatment approaches that address the unique needs of LGBT + individuals diagnosed with SMI.

**Table 1:** Summary table of the included studies

Authors	Year	Pupulation	Demographics	Methods	Key Findigs
Pehlivan et al.	2022	One hundred and seven young people (18–25 years) participated and of these, 37.7% were inpatients.	One hundred and seven young people (18–25 years). Mean age 21.9 (± 1.9); 79.4% Australian-born. Gender: 92.5% cis-male/cis-female, 7.5% gender-diverse. Sexual identity: 61.7% heterosexual, 38.3% other. Diagnoses: 64.5% psychotic disorder, 17.8% personality disorder, 17.8% mood/anxiety disorder. 46.7% had co-morbid substance use disorder.	Cross-sectional survey on sexual health among youth mental health service outpatients/inpatients, analyzing statistical differences between groups.	Inpatients were less likely to have regular sexual partners (25% vs. 64.5%) and more likely to use amphetamines during sex (28.6% vs. 5.8%) compared to outpatients. Sexual dysfunction was higher in inpatients (55.6% vs. 37.9%). High-risk behaviors and dysfunction were prevalent in both groups, emphasizing the need for integrated mental, physical, and sexual health services.
de Jager et al.	2017	Twenty-eight participants with a psychotic disorder.	Between 18 and 65 years of age (mean = 42 years) from four community mental health teams specializing in flexible assertive community treatment.	Semi-structured interviews on intimate relationship needs and experiences, transcribed and analyzed using Grounded Theory methodology.	Five key barriers to intimate relationships: medication side effects, mental symptoms, stigma/self-stigma, sexual abuse, and lack of social skills/experience. Loss of self-esteem was a central theme across all categories.
Anex et al.	2023	Inpatients primarily diagnosed with a mental health disorder.	Patients aged ≥ 18.	Qualitative systematic review.	The review identified five themes: (1) policies/guidelines, (2) MHPs’ and inpatients’ opposing attitudes (safety vs. human rights), (3) impact/strategies, (4) barriers, and (5) facilitators. Most studies reported implicit staff norms, with limited policy documents available. MHPs viewed inpatient sexual behavior as unsafe, while inpatients emphasized it as a human right. Trends in non-European countries and capacity to consent in the context of human rights were discussed. Policy impact assessment was limited due to insufficient documentation.
Hache-Labelle et al.	2021	Twenty-nine young men with early psychosis.	Heterosexual single man aged between 18 and 30.	Cognitive behavioural group intervention occurring over 12 sessions and focusing on romantic relationships.	Feasibility and acceptability confirmed. Mixed intervention impact: improvements in social functioning (behaviors), romantic relationships, and Theory of Mind (mentalizing). Further research needed to support social and romantic development in men with early psychosis.
Stusiński et al.	2022	People with SCH	No specific demographics provided.	Narrative review of the literature.	Dyadic relationships are vital for quality of life, including for individuals with schizophrenia (SCH). Desire for close relationships can motivate health improvement. Challenges include illness symptoms, social stigma, and cognitive deficits, with gender differences favoring women in relationship formation. Cognitive deficits and emotional expression difficulties hinder relationships, leading to high separation rates. Successful relationships support therapy adherence and functional remission, highlighting the need to integrate relationship support into SCH treatment.
Hortal-Mas et al.	2022	People who suffer a serious mental illness	No specific demographics provided.	Meta-synthesis to integrate qualitative studies.	Four categories identified: (1) “Pathologized sexuality” (impact of disorder/treatment), (2) “Not my sexuality anymore” (self-acceptance challenges), (3) “Learning to manage intimate relationships” (desire for meaningful connections), and (4) “Reconstructing my sexuality” (environmental influence). Sexuality is affected by clinical complications, medication side effects, social support, healthcare relationships, and stigma. Serious mental illness can lead to suffering and isolation, but individuals maintain interest in an active sex life. Mental health services should address sexual health, reduce stigma, and train nurses in affective/sexual education to support patients.
Yang et al.	2023	20 patients with schizophrenia.	Aged 18–60 (range 18–40 70%), diagnosed with schizophrenia by a psychiatrist using ICD-10/DSM-5 criteria, and with stable symptoms (PANSS score change < 15 and total score < 60 in the past 4 weeks).	Face to face semi-structured in-depth interviews, qualitative study using a descriptive phenomenological approach.	Three macro themes emerged: (1) multiple barriers to sexual activity, (2) significance of sex, and (3) conditions for fulfilling sexual needs. Patients with schizophrenia often experience poor sexual quality of life but retain interest in an active sex life. Mental health services should address sexual knowledge, space, and objects to support patients.
Cloutier et al.	2023	196 people diagnosed with a schizophrenia-spectrum disorder.	18 years of age or older people (mean = 35.78 years) recruited and self-referred from several clinics specializing in psychosis.	Online questionnaires (Multidimensional Sexuality Questionnaire and the Romantic Relationship Functioning Scale).	People with severe mental illness showed low social functioning, particularly in independent living, relationships, and work/academic performance. Intimate relationships and sexuality were especially challenging, with difficulties in dating, social skills, attachment (fear of proximity/abandonment), and self-esteem, despite maintaining friendships and family ties.
Barker et al.	2023	19 individuals with early psychosis	Aged 18–31 years, cisgender and transgender women and non-binary individuals, receiving clinical care from early psychosis programmes in Ontario, Canada.	Semi-structured individual qualitative interviews.	Young women and non-binary individuals with early psychosis reported diverse sexual health experiences, including changes in sex drive, vulnerability during acute psychosis, and bidirectional mental health-relationship links. These were influenced by identity, autonomy, gender, and sexual orientation. Clinicians prioritized risk prevention, while patients emphasized holistic sexual health. Integrated clinical care is needed to address their diverse sexual health priorities and optimize well-being.
White et al.	2023	190 people with lived experience of psychosis	Participants aged ≥ 16 with a schizophrenia spectrum diagnosis, psychosis-related mental health support, psychiatric inpatient stays for psychosis, or antipsychotic medication use.	Online survey measuring relationship satisfaction (Satisfaction with Relationship Scale), psychotic symptoms (CAPE-42), and general well-being (Short Warwick-Edinburgh Mental Wellbeing Scale).	Fearful attachment and partner criticism reduced relationship satisfaction, while having a partner increased it. Higher relationship satisfaction correlated with lower psychotic symptoms and better mental well-being, mediated by loneliness, stigma, and self-esteem. Mental health services should address relationship satisfaction, especially for those with fearful attachment styles.
de Jager and McCann.	2017	People diagnosed with schizophrenia or related psychosis.	No specific demographics provided.	Literature review qualitative studies.	People with psychosis report unsatisfactory sexuality and intimacy, with needs similar to the general population. Preventive interventions targeting sexual risks are needed. Sexual dysfunction due to psychotropic side effects is widely studied, with ~ 25% facing prejudice and self-stigma, leading to social withdrawal and reduced functioning. Sexual fantasies resemble those of healthy individuals, though sexual obsessions may be more frequent. Psychosis can blur sexual orientation/gender identity, causing confusion or shame. Limited research on LGB individuals link minority stress to psychotic symptoms. Childhood sexual adversities and post-psychosis victimization are significant risks, with impaired consent capacity playing a role.
Grachev et al.	2022	Populations living with serious mental illness.	Adult women (18 years +) with a DSMI that meets the diagnostic criteria of DSM-IV/V.	Qualitative literature review using PRISMA.	Women with DSMI face unique barriers to sexual fulfillment due to stigma, gendered socialization, interpersonal relationships, and psychiatric institutions. These barriers operate at subjective, interpersonal, and structural levels, perpetuating oppression, diminished self-worth, social withdrawal, and control over their sexuality by dominant systems.
Gerymski and Szelag.	2023	60 individuals with schizophrenia (31 women and 29 men).	Participants aged ≥ 18 (mean = 31.27) from Poland.	Self-Esteem Scale (SES), Acceptance of Illness Scale (AIS), and Short Sexual Well-Being Scale (SSWBS). Parametric analyzes were chosen to analyze the data.	Self-esteem and acceptance of illness significantly predicted sexual well-being in individuals with schizophrenia (β = 0.62 and β = 0.55, respectively). Acceptance of illness mediated the relationship between self-esteem and sexual well-being (indirect effect: β = 0.34). Findings emphasize the importance of acceptance of illness in maintaining sexual well-being in schizophrenia.
Barchielli et al.	2022	46 patients with a diagnosis of acute psychosis or schizophrenia, in addition 52 healthy control subjects.	Participants aged ≥ 18 (mean for males = 41.20 years, for females 46.28 years).	Data Analysis.	Psychotic individuals, both male and female, showed significant differences in sexual functioning compared to healthy controls, with higher sexual dysfunction and lower quality of sexual life. Assessment and support for sexual function in psychotic patients are essential to address these issues and improve their quality of life.
Brown et al.	2023	69 young people with FEP were included in this study.	Young people (15–24 years) with FEP attending the EPPIC service in Melbourne.	Data Analysis.	78.3% of the cohort had been sexually active, with low rates of consistent condom (44.2%) and barrier contraception (35.7%) use. 22.5% had prior pregnancies, and 18.6% tested positive for STIs. Substance use increased sexual activity. High-risk sexual behaviors are prevalent in first-episode psychosis, highlighting the need for early interventions to promote sexual well-being and communication skills.
Vickers et al.	2022	Young people with psychosis.	Young people (aged 14–24 years) with psychotic disorders.	Systematic scoping review.	Sexual dysfunction is common in psychosis, linked to both the disorder and antipsychotics. This population engages in higher sexual risk-taking and has increased STI risk. Key SRH topics (e.g., pregnancy, sexual violence, identity) are understudied in younger patients. Empirical studies lacked confounder control, and case reports provided limited outcome descriptions post-intervention.
Budziszewska et al.	2020	10 persons diagnosed with schizophrenia.	5 women and 5 men (mean age of participants was 34.4) experienced severe mental illness diagnosed with schizophrenia.	Qualitative interpretative phenomenological analysis (IPA) of the semi-structured interviews.	Illness and hospitalizations alienate relationships through stigma and self-stigma, creating psychological barriers like diminished trust. Long-term patients face practical challenges (e.g., poverty) in forming and maintaining relationships. Patients report changes in sexuality and associated risks, offering insights into coping strategies from their perspectives.
Adan Sanchez et al.	2019	103 young people patients of the of OYH.	Aged between 15–25 diagnosed with ultra-high risk for psychosis, first episode psychosis.	Cross-sectional survey.	Young people with a mental health disorder in this study had a high prevalence of high-risk sexual behaviours and their sequelae. These high-risk sexual behaviours were homogeneous across specialist clinics and psychiatric symptomatology. Additionally, there was a high incidence of pregnancy, STIs and inconsistent contraceptive use.
Schonewille et al.	2022	Women with a psychiatric diagnosis at time of conception and reported pregnancy intention.	Women with age ≥ 18.	Systematic review and meta-analysis.	Women with psychiatric vulnerabilities (e.g., mood, anxiety, psychotic, substance use, conduct, and eating disorders) have a higher risk of unintended pregnancies (UPs) (OR 1.34, CI 1.08–1.67) and a weighted up prevalence of 65% (CI 0.43–0.82). Research on this topic is limited, with fair-to-poor study quality due to inconsistent pregnancy intention measurement and lack of control groups. Findings highlight the need for family planning discussions in this population to mitigate risks for mother and child.
de Jager et al.	2018	Twenty-eight participants with a psychotic disorder.	Aged 22 to 62 years (mean = 42 years)	Twenty-eight semi-structured interviews analyzed using the Grounded Theory method.	Almost all participants experienced unfulfilled needs in sexual expression. These unfulfilled needs were associated with a range of factors, including antipsychotic medication, psychotic symptoms, sexual abuse, social skills and stigma, all of which may converge on a pathway involving (sexual) self-esteem. Further research is required to better understand the role of self-esteem in relation to sexual needs and expression in people with psychotic disorders.
McCann et al.	2019	People diagnosed with SMI.	Above 18 years old who have been diagnosed by a clinician with SMI.	Qualitative systematic review.	This article provides valuable insights into the sexual and intimacy-related challenges faced by people with SMI. The sexuality of individuals with SMI is shaped by complex, individualized experiences, often hindered by internalized and external stigma. Barriers to intimacy emerge from both self-stigma and societal perceptions, limiting the formation of meaningful relationships. Several studies highlighted sexual vulnerability and coercion within this group, with such experiences sometimes acting as lifelong obstacles to sexual expression. Additionally, altered perceptions of sexuality during psychosis necessitate careful interpretation, as ambiguous or unclear expressions should not be dismissed as purely delusional. Despite a strong desire for intimacy, individuals with SMI face significant challenges in achieving fulfilling relationships.
Kaplan et al.	2022	13 men diagnosed with schizophrenia, psychotic disorders, or bipolar disorder.	Above 18 years old (61,54% age range 32–46).	Semi-structured interviews.	Two main themes emerged: (1) Challenges in expressing sexuality in psychiatric wards, including limited space, medication side effects, staff dialogue barriers, and perceived boundary violations; and (2) Patient-suggested improvements, such as open staff dialogue, access to protected sex measures, and policy changes. Findings underscore the lack of healthy sexual expression in wards, highlighting the need for staff awareness, national policies, and tailored solutions to address patients’ sexual needs and improve well-being
Fanta et al.	2018	422 patients with schizophrenia.	Age group 18 and above (range 18–24 8,3%; 25–34 years, 41.2%; 35–44 years 35,5%; >45 years 14,9%).	Hospital based cross sectional study.	The magnitude of Sexual dysfunction was found to be high among patients with schizophrenia and it is associated with different factors like unmarried, divorced, widowed, relapse and poor quality of life. Treating physicians should be conscious to sexual dysfunction during evaluation and treatment of patients with Schizophrenia. Special attention should be given to single, divorced, widowed patients and patients with history of relapse to improve quality of life of this patient.
Jones et al.	2018	49 people treated for a psychotic disorder.	Average age 39 years (range 18–67 years).	In-depth phenomenological interviews and	Sex/gender-related content was reported by ~ 75% of interview and 25% of focus group participants. Themes included shame/persecution, sexual violence, power/agency/sexuality/gender, and positive erotic experiences. Shame was common, with sexual content being primary or secondary in experiences. Clinically, validating and engaging with these themes is crucial, warranting further research on treatment and social recovery implications.

## Discussion

Numerous research studies have shown that people struggling with severe mental illness often have unrecognized and unmet needs for intimacy, love and sexuality [5, 12, 27]. Despite clear evidence indicating that their desires, fantasies and desires for romantic relationships are closely aligned with those of the general population, these needs are often overlooked and remain unaddressed [12, 17].

As far as serious psychiatric illness is concerned, it is evident that the greatest risk for the onset of risky sexual behaviour occurs for individuals who demonstrate sufficient social and personal skills to establish and maintain relationships with others and thus entertain sexual relationships. For this reason, more adolescents and young adults, those subjects, therefore, in whom the psychiatric illness is at its first onset or, at any rate, not yet chronic [27, 29, 30, 33].

On the other hand, for more adult subjects or for those whose course of the illness is more advanced, there seems to be a greater propensity for isolation and victimization, which excludes them from the possibility of establishing meaningful emotional relationships and maintaining sexual well-being [28, 36]. This is attributable both to the social stigma that afflicts psychotic subjects and to the symptoms of the clinical condition itself, which make it complex to interact and maintain intimate relationships with others and which, in association with side-effects of medications, gender differences and certain personal dimensions, such as poor acceptance of the illness and low self-esteem, contribute to the isolation and lack of sexual well-being that characterize this population [9, 26, 32, 37].

Most of the studies identified in this review agree that being able to count on a healthy and satisfying sexual and intimate life is a fundamental element for the recovery of patients with severe psychiatric illness because there is an increase in some fundamental psychological dimensions, such as self-esteem and self-efficacy [16, 17]. Moreover, it is evident from the various studies that sexual and affective well-being leads to an improvement in family and emotional relationships as well as better compliance with pharmacological and therapeutic treatments [16, 17].

### Implications for professional practice and recommendations

The findings from this review highlight the critical need for more inclusive, individualized approaches within professional practice to support the intimate and sexual well-being of young adults and adolescents with severe mental illness. Given the unique challenges faced by these populations, professionals across mental health, social work, education, and healthcare settings should consider a few key recommendations. These include the promotion of tailored sexual education, the integration of relational needs into care planning, the reduction of stigma, the adoption of strength-based approaches, the development of inclusive policies and training, the creation of LGBTQ+-affirming environments, and the long-term support of relationship skills. Table 2 provides a summary of these seven domains, each of which is explored in greater detail below, along with practical considerations for their implementation across care settings:

1. Incorporate Comprehensive Sexual Education programs. Sexual education tailored to the cognitive and emotional needs of young people with severe mental illness is essential. Programs should include safe practices, consent, boundaries, and managing relationships, ideally through accessible materials and consistent reinforcement. Early, developmentally appropriate interventions can prevent risky sexual behaviors and reduce vulnerability to exploitation;
2. Integrate support for romantic and intimate relationships in treatment plans. Professionals should view romantic relationships and sexual expression as integral to the holistic well-being of individuals with these diagnoses. Practitioners could facilitate social skills training, self-esteem enhancement, and interpersonal communication to help these individuals build healthy, fulfilling relationships. Where possible, family members and caregivers should be included in these discussions to foster a supportive environment;
3. Address social stigma in therapeutic contexts. Social stigma significantly affects individuals’ self-perception and their perceived eligibility for romantic relationships. Mental health providers should actively counter stigma by encouraging self-acceptance and fostering a positive identity beyond the diagnosis. Group therapy or peer-support groups focusing on relationship-building might help reduce isolation and promote self-confidence;
4. Adopt a person-centered, strength-based approach. Emphasizing strengths over deficits can empower individuals to express their desires and pursue relationships in a safe, supported context. For instance, encouraging clients with SMI to explore personal resilience and relational strengths may facilitate their engagement in intimacy and improve self-worth;
5. Develop inclusive, evidence-based policies and training. Organizations should provide training that sensitizes staff to the unique needs of individuals with severe mental illnesses in areas of intimacy and sexual health. Practical guidance for managing complex scenarios, like sexual behaviors in communal settings, will help professionals respond effectively and compassionately;
6. Create safe spaces for LGBTQ + individuals with SMI. LGBTQ + individuals within these populations often face dual stigma, impacting their well-being and access to appropriate support. Professionals should create inclusive environments that affirm diverse gender identities and sexual orientations, addressing the compounded discrimination these individuals may face in both medical and social contexts.
7. Support long-term relationship skills development. Given the lifelong impact of disabilities and severe mental health conditions, providing ongoing support for relationship skills, including navigating the end of relationships, is essential. Regular follow-ups and adaptive interventions will address the evolving needs of clients as they age.

Incorporating these recommendations into professional practice can significantly improve the quality of life for young people with severe mental illness by reducing risks and fostering greater autonomy in their relationships. By addressing these essential aspects of well-being, professionals not only enhance individual health outcomes but also affirm the fundamental rights of these individuals to experience intimacy, love, and self-expression, core elements of a person-centered approach in mental health care.

**Table 2:** Summary of key recommendations for practice

	Domain	Key Focus
1	Tailored Sexual Education	Developmentally appropriate content on consent, safety, and relationships
2	Romantic Relationship Support	Inclusion of intimacy and love in treatment plans
3	Reducing Social Stigma	Promote self-acceptance and counter stereotypes in clinical settings
4	Strength-Based Approaches	Focus on resilience, capacities, and subjective agency
5	Inclusive Training and Policies	Professional education and protocols on sexual health and intimacy
6	Affirming LGBTQ + Environments	Address dual stigma and ensure inclusive, supportive spaces
7	Long-Term Relational Skill Development	Ongoing support for autonomy and adult relational life

### Limits of the study

This literature review has several limitations that should be acknowledged. First, the inclusion criteria for the studies were restricted to English-language publications, which may have led to the exclusion of relevant studies published in other languages. This restriction could limit the comprehensiveness of the review and the diversity of perspectives included. Second, the review focused primarily on literature published between 2017 and 2023, which may not fully capture earlier significant studies that could provide valuable insights into the sexual health and relationship dynamics of individuals with severe mental illness. Consequently, this temporal limitation may overlook historical trends and foundational theories in the field. Moreover, while the studies reviewed aimed to prioritize the perspectives of young individuals themselves, the inherent challenges in effectively capturing their voices through existing research may result in an incomplete representation of their experiences and needs. Most of the articles included in this review relied on self-reported data, which may not fully capture participants’ own perspectives on their sexuality. Instead, the quantitative data reflect the aspects of sexuality that researchers chose to investigate and report. For this review, findings were organized according to common categories related to sexuality, based on these reported data, which may not encompass the full range of participants’ personal experiences and insights. Additionally, the scope of the review was limited to specific populations diagnosed with severe mental illness (schizophrenia and psychotic disorders). This focus might exclude critical insights from other related groups, such as those with different psychiatric diagnoses, who may also face similar challenges in their sexual and relational lives. We also acknowledge that the initial search strategy included the term “Down syndrome”; however, studies focusing on this population were excluded from the final analysis to ensure conceptual coherence and consistency with the defined scope of the review. Lastly, the review’s findings are constrained by the variability in definitions and measurements of sexual health and intimacy across different studies, which can complicate comparisons and generalizations. This lack of standardized terminology may lead to inconsistencies in understanding the experiences of these individuals. Future research should address these limitations by expanding the scope of inquiry, employing diverse methodologies, and incorporating broader populations to enrich the understanding of sexuality and intimacy among individuals with severe mental illness.

## Conclusions

This review highlights that while adolescence and young adults with severe mental illness express a need for intimacy and romantic connection similar to that of their peers, they face unique barriers that frequently leave these needs unmet. Social stigma, alongside the effects of severe psychiatric symptoms, contributes to the isolation and vulnerability of this population, with stigma often impacting both self-perception and social interactions. The review findings suggest that acknowledging and addressing these relational needs can support overall well-being, though further research is necessary to fully understand the complex dynamics of intimacy and romantic relationships in individuals with severe mental illness. It is important to note the limitations of this review, including its reliance on a varied body of literature that may not uniformly represent all individuals with severe mental illness. Additionally, much of the existing research focuses on heterosexual relationships, limiting the applicability of findings to LGBTQ + individuals. The absence of long-term, longitudinal studies on intimacy and romantic relationships in this population further restricts our understanding of how these needs evolve over time and interact with ongoing treatment. Finally, direct input from individuals with severe mental illness was limited within the reviewed studies, highlighting the need for future research that prioritizes their personal experiences and perspectives.

## Supplementary Information

Below is the link to the electronic supplementary material.

Supplementary Material 1

Supplementary Material 2

## Data Availability

All data generated or analysed during this study are included in this published article [and its supplementary files 1 and 2].

## Abbreviations

IEP: Individual with Early Psychosis
DSMI: Diagnosed with Severe/Serious Mental Illness
PLSMI: People Living with Serious/Severe Mental Illness
SCH: Schizophrenia
SMI: Severe/serious Mental Illness
