# Supplementary material for: Romantic relations, sexuality and intimacy among young adults and adolescents with severe mental illness: a review of the literature
Source: BMC Psychiatry. 2025 Nov 26;25:1193. doi: 10.1186/s12888-025-07224-1 (PMC12750685; doi:10.1186/s12888-025-07224-1)
Supplement: Supplementary file 1 — Supplementary Material 1 [file 12888_2025_7224_MOESM1_ESM.pdf]

Additional file 2: Search strategy. Full search strategies for Scopus and Web of Science

Search strategy: Scopus

1) TITLE-ABS-KEY ("Down syndrome" OR schizophrenia OR psychosis OR "mental disability" OR "mental illness") AND (adolescence OR "young adult" OR "young adults" OR "teenager") AND (sexuality OR intimacy OR love OR romance OR romantic)
2) Filters: (LANGUAGE: English, DOCUMENT TYPE: article and review, YEAR: range 2017-2023)

Search strategy: Web of Science

1) TOPIC ("Down syndrome" OR schizophrenia OR psychosis OR "mental disability" OR "mental illness") AND (adolescence OR "young adult" OR "young adults" OR "teenager") AND (sexuality OR intimacy OR love OR romance OR romantic)
2) INDEX DATE (2017-01-01 to 2023-12-31)
3) REFINED BY: (DOCUMENT TYPES: Review article or Article) and (LANGUAGES: English).
