# Supplementary figures and images for: Romantic relations, sexuality and intimacy among young adults and adolescents with severe mental illness: a review of the literature
Source: BMC Psychiatry. 2025 Nov 26;25:1193. doi: 10.1186/s12888-025-07224-1 (PMC12750685; doi:10.1186/s12888-025-07224-1)

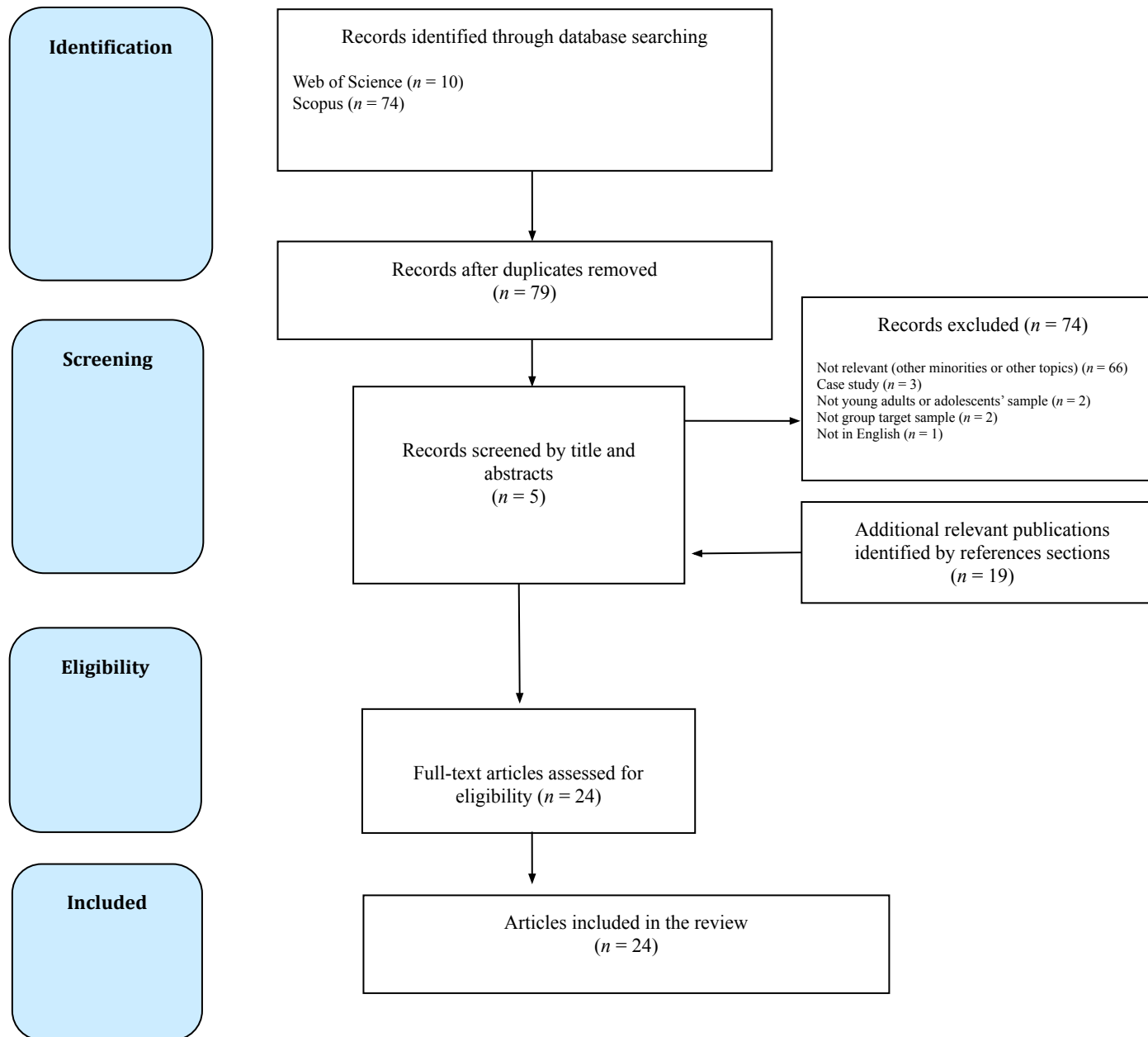

Supplement: Supplementary file 2 — Supplementary Material 2 [file 12888_2025_7224_MOESM2_ESM.pdf]
